# α-Synuclein gene expression profile in the retina of vertebrates

**Published:** 2007-06-18

**Authors:** Gema C. Martínez-Navarrete, José Martín-Nieto, Julián Esteve-Rudd, Antonia Angulo, Nicolás Cuenca

**Affiliations:** 1 Departamento de Fisiología, Genética y Microbiología, Facultad de Ciencias, Universidad de Alicante, Alicante, Spain; 2 Departamento de Optica, Farmacología y Anatomía, Escuela Universitaria de Optica y Optometría, Universidad de Alicante, Alicante, Spain

## Abstract

**Purpose:**

α-Synuclein is a Parkinson's disease-linked protein of ubiquitous expression in the central nervous system. It has a proposed role in the modulation of neurotransmission and synaptic function. This study was aimed at analyzing expression of the α-synuclein gene in the normal retina, and characterizing its pattern of distribution in the different retinal cell types and layers in a variety of vertebrates, ranging from fish to humans.

**Methods:**

Reverse transcriptase-polymerase chain reaction and immunoblotting were used to assess α-synuclein expression at both mRNA and protein levels. Its retinal distribution profile was characterized by immunohistochemical methods. With this purpose, retinal sections were analyzed under fluorescent confocal microscopy using specific antibodies against α-synuclein, alone and in double or triple combinations with a set of antibodies to molecular markers for the distinct retinal neuronal types. Also, synaptophysin was used as a marker for synaptic vesicles in the retina.

**Results:**

α-Synuclein mRNA and protein were expressed by both retinal pigment epithelium (RPE) and neural retinal cells. The pattern of α-synuclein distribution in the retina was quite consistent across all vertebrate species examined. A strong immunoreactivity was found in the outer segments (OS) of photoreceptors and in their axon terminals (cone pedicles and rod spherules) in the outer plexiform layer (OPL) of the retina. α-Synuclein was also present in rod and cone bipolar cells, as well as in GABAergic and glycinergic amacrines, distributing along a complex plexus throughout the inner plexiform layer (IPL). Additionally, colocalization was found between α-synuclein and synaptophysin at presynaptic terminals of the retina. α-Synuclein-positive phagosome-like structures were observed in the cytoplasm of RPE cells.

**Conclusions:**

An involvement of α-synuclein can be postulated in neurotransmission at axon terminals of photoreceptors in the OPL, and at presynaptic endings of bipolar and amacrine cells in the IPL. As well, this protein could have a role in the function as well as the maintenance of photoreceptor OS. α-Synuclein contained in RPE cells should derive not only from protein expression by this cell type, but also from their phagocytosis of OS disc membranes.

## Introduction

α-Synuclein is a highly-conserved 140 amino-acid neuronal protein expressed in numerous areas throughout the brain [1-5] and enriched in presynaptic terminals [2,3,6,7]. A number of roles have been ascribed to α-synuclein in synaptic function, maintenance, and plasticity in the central nervous system (CNS) [7-10]. High levels of this protein are found in midbrain dopaminergic neurons [5,11,12], where it appears to modulate nigrostriatal neurotransmission and tyrosine hydroxylase activity [13-15]. Mutations in the α-synuclein-encoding gene, *SNCA* (OMIM 163890), have been associated with familial autosomal-dominant, juvenile forms of Parkinson's disease (PD), which are phenotypically equivalent in their clinical symptoms to sporadic parkinsonism. α-Synuclein is known to constitute the major fibrillar component of Lewy bodies [16], i.e. the large intracytoplasmic inclusions typically observed within dopaminergic neurons of idiopathic and familial PD patients. The colocalization of α-synuclein and ubiquitin in these inclusions has led to the hypothesis that conformational changes derived from mutation or overexpression of the α-synuclein gene hamper its degradation by the ubiquitin-proteasome system, with ensuing cytosolic accumulation, aggregation and fibrillogenesis [10,15,17,18]. Accumulation of wild-type α-synuclein has also been found with synaptic loss in the substantia nigra of monkey models of PD treated with the neurotoxic 1-methyl-4-phenyl-1,2,3,6-tetrahydropyridine (MPTP), which selectively kills dopaminergic cells of the brain and the retina [19,20]. α-Synuclein deposits have also been reported in patients with other neurodegenerative disorders involving Lewy body formation, collectively known as "synucleopathies", including dementia with Lewy bodies, multiple system atrophy, amyotrophic lateral sclerosis, and some Alzheimer's disease variants [7,8,10,21,22]. Furthermore, transgenic overexpression of human wild-type or mutant α-synuclein variants in the mouse brain leads to loss of dopaminergic neurons and decrease of tyrosine hydroxylase levels, correlating with the formation of Lewy body-like intraneuronal inclusions and the development of motor deficits [23-26].

Apart from studies in the brain, the expression and distribution of α-synuclein in the normal retina of vertebrates has not been studied in depth. Given the growing body of experimental evidence concerning visual dysfunction and morphological impairments in the retina of PD patients and MPTP-treated animals [27-30], including that from our group [20], we set out to analyze α-synuclein expression at the mRNA and protein levels and to characterize its distribution pattern in the distinct retinal layers and cell types. This study involved a wide spectrum of vertebrates, including both non-mammalian and mammalian species ranging from fish to humans, and was designed to obtain clues to the physiological role of α-synuclein in the retina.

## Methods

### Biological material

The following non-mammalian vertebrate species were studied in this work: carp (*Cyprinus carpio*), African clawed toad (*Xenopus laevis*), yellow-bellied slider turtle (*Trachemys scripta scripta*), and chicken (*Gallus gallus domesticus*). The following mammalian species were studied: mouse (*Mus musculus*, C56BL/6J unless otherwise stated), rat (*Rattus norvegicus*, Wistar), cat (*Felis catus*), California ground squirrel (*Spermophilus beecheyi*), European wild rabbit (*Oryctolagus cuniculus*), Iberian pig (*Sus scrofa mediterraneus*), cow (*Bos taurus*), cynomolgus monkey (*Macaca fascicularis*), and human (*Homo sapiens*). Carps and turtles were purchased from local petshops. Xenopus, rodents, and cats were kept at our university animal-care facilities. Monkeys (five to six years old) were kept at the Universidad de Murcia in accordance with the International Primate Society guidelines. They were maintained in a special room with controlled temperature (22-24 °C), a 12 h light and dark cycle, humidity at 40-60% and suitable ventilation [31]. They were given fresh fruit and special food for primates (Old World Primate Diet Expanded) from B & K Universal (Hull, UK), and provided with water ad libitum. No individual was killed solely for the purpose of the research reported here, but instead other body parts were harvested by different researchers for a range of unrelated experiments.

All animal handling was carried out in compliance with the guidelines set by the National Institutes of Health and the European Directive 86/609/EEC. Animals were euthanized according to standard, internationally-approved protocols, and their eyes were immediately enucleated afterwards. Fresh chicken, rabbit, porcine, and bovine eyes were provided by local farms or slaughterhouses. Paraformaldehyde-fixed squirrel eyes were obtained from the University of California (Santa Barbara, CA). Post-mortem human eyes from donors in the 45-60 year-old range were obtained from the Utah Lions Eye Bank (Salt Lake City, UT). For RNA and protein extraction, dissected retinal fractions were snap-frozen in liquid nitrogen and immediately stored at -80 °C without fixation. For immunohistochemistry, eyes were fixed in 4% paraformaldehyde in 0.1 M sodium phosphate buffer pH 7.4 for 1 h, and then subjected to sucrose cryoprotection [32] before storage at -80 °C.

### Reverse transcriptase-polymerase chain reaction

RNA was extracted from the neural retina and retinal pigment epithelium (RPE) fractions using the RNAqueous-4PCR Kit from Ambion (Austin, TX), and then treated with DNAse I following the manufacturers' instructions. RNA from human retina was purchased from Clontech BD (Mountain View, CA). Reverse transcription into cDNA was carried out using the RETROscript Kit (Ambion), at 44 °C for 1 h in 20 μl of a reaction mixture containing 0.5-1.0 μg of RNA, the four dNTPs at 0.5 mM each, 5 μM oligo(dT) or random decamers, 10 U of RNase inhibitor and 100 U of MMLV reverse transcriptase. PCR amplification was carried out from 10 ng of cDNA in 50 μl of a reaction mixture containing forward and reverse primers at 0.25 μM each, the four dNTPs at 125 μM each, and 1 U of *Taq* DNA polymerase (Amersham Biosciences, Buckinghamshire, UK). Using the Primer3 software [33], we designed PCR primers to bear a T_m_ of 60 °C, flank at least one intron, and have 100% identity with their corresponding cDNA sequences. The primers were as follows: for human and monkey α-synuclein (Genbank NM_000345 and AH013262, respectively), forward: 5'-AAA ACC AAG GAG GGA GTG GT-3' (exon 3), reverse: 5'-TCA AGA AAC TGG GAG CAA AGA-3' (exon 6); for bovine α-synuclein (Genbank NM_001034041), forward: 5'-GTG GTG ACA GGG GTG ACT G-3' (exon 4), reverse: 5'-CTT CGG GTT CGT AGT CCT GA-3' (exon 6); for mouse and rat α-synuclein (Genbank NM_009221 and NM_019169, respectively), forward: 5'-GCA GTG GTG ACT GGT GTG AC-3' (exon 4), reverse, 5'-CGA TCA CTG CTG ATG GAA GA-3' (exon 6). After an initial denaturation step at 95 °C for 5 min, amplification was performed for a total of 30 cycles each at 60 °C for 1 min, 72 °C for 2 min, and 95 °C for 1 min, and concluded with a final annealing at 60 °C for 1 min and elongation at 72 °C for 5 min. PCR products were run on 2% agarose gels stained with SYBR Green I (Sigma Chemical, St. Louis, MO) and verified by automated DNA sequencing.

### Immunoblotting

Retinal proteins were extracted following guidelines described in reference [34]. Briefly, cells were lysed for 15-20 min on ice in two volumes of a buffer composed of 20 mM sodium Hepes pH 7.9, 10% glycerol, 10 mM KCl, 0.4 M NaCl, 1% Nonidet P-40, 2 mM DTT and protease inhibitors (1 mM PMSF, 10 μM leupeptin, 0.3 μM aprotinin, and 100 μM benzamidine). The supernatants obtained after centrifugation at 16,000xg for 10 min were used for immunoblotting analysis. Proteins (50 μg/lane) were resolved by SDS-PAGE on 5-20% polyacrylamide-gradient gels, and after electrotransfer to PVDF membranes (Hybond-P, Amersham) stained with SYPRO Ruby Protein Blot Stain (Molecular Probes, Eugene, OR) to ensure even protein loading and transfer. Membranes were then probed sequentially with rabbit polyclonal antibody to α-synuclein (Chemicon AB5038, Temecula, CA) at a 1:2000 dilution overnight and horseradish peroxidase-conjugated anti-rabbit IgG antibody at a 1:1000 dilution for 1 h. They were developed by means of enhanced chemiluminescence (ECL) using the ECL plus Western blotting detection system kit (Amersham). Images were obtained upon exposure of Hyperfilm ECL plates to the blots.

### Immunohistochemistry

Cryostat vertical 16 μm retinal sections were obtained and processed for immunohistochemistry based on guidelines established in references [20,32,35]. All primary antibodies used in this work (summarized in Table 1) have been utilized in several previous studies and are well characterized by us and other authors regarding specific cell-type immunostaining. Sections were subjected to single, double or triple immunostaining overnight at room temperature with antibodies against α-synuclein and a set of molecular markers for the distinct retinal neuronal types, at the dilutions indicated in Table 1 in 0.1 M sodium phosphate buffer pH 7.4 plus 0.5% Triton X-100. Thereafter, the corresponding secondary antibodies to IgG (Molecular Probes) conjugated to Alexa Fluor 488 (green), 546 (red), or 647 (far red) were applied at a 1:100 dilution for 1 h. Control slides in which primary antibodies were omitted were processed in parallel, and no immunoreactivity was found in any case. Fluorescence was detected with a TCS SP2 confocal laser-scanning microscope (Leica Microsystems, Wetzlar, Germany), using a pinhole diameter of 77 μm. Images were obtained sequentially from the green, red and/or far-red channels as optical slices with a thickness <0.9 μm. Images from the far-red channel were pseudocolored in blue.

**Table 1:** Primary antibodies used for immunohistochemistry.

**Molecular marker**	**Antibody**	**Source**	**Working dilution**
α-Synuclein	Goat polyclonal	Santa Cruz Biotechnology (Santa Cruz, CA), sc-7011	1:100
Calbindin D-28K	Mouse, clone CB-955	Sigma Chemical, C9848	1:500
GABA	Rat polyclonal	D.V. Pow, University of Queensland (Brisbane, Australia)	1:300
Glycine	Rat polyclonal	D.V. Pow	1:300
Microtubule-associated protein-2 (MAP2)	Rabbit polyclonal	Chemicon, AB5622	1:500
Protein kinase C, α isoform (PKCα)	Mouse, clone MC5	Santa Cruz Biotechnology, sc-80	1:100
Recoverin	Rabbit polyclonal	J.F. McGinnis, University of Oklahoma (Oklahoma City, OK)	1:5000
Rhodopsin	Mouse, clone 4D2	R.S. Molday, University of British Columbia (Vancouver. Canada)	1:100
S-cone opsin	Mouse, clone JH455	J. Nathans, Johns Hopkins School of Medicine (Baltimore, MD)	1:500
Synaptophysin	Mouse, clone SY38	Chemicon, MAB5258	1:500
Transducin, Gγc sub-unit	Rabbit polyclonal	Cytosignal (Irvine. CA), PAB-00801-G	1:500

Recorded are the primary antibodies used in this study. The antibody column lists antigen and animal origin. The clone designation is given for monoclonal antibodies. The source column provides either the commercial company and antibody reference, or the investigator and affiliation. The last column specifies the dilution at which each antibody was used on retinal sections.

## Results

**Figure 1:**
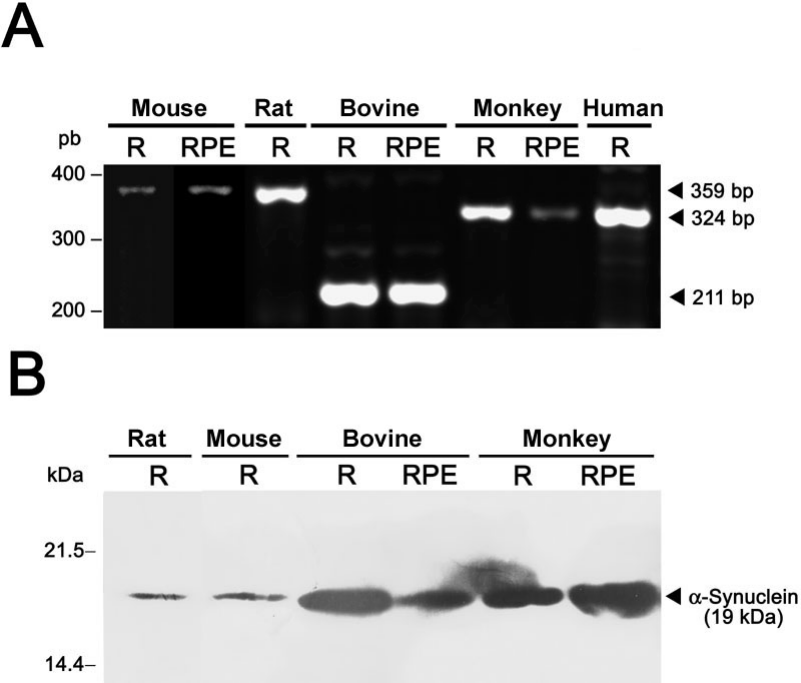
Expression of the α-synuclein gene in the retina of mammals. Reverse transcriptase-polymerase chain reaction (**A**) and immunoblotting analysis (**B**) of α-synuclein mRNA and protein expression, respectively, were carried out in the neural retina (R) and retinal pigment epithelium (RPE) fractions from the indicated species. Molecular sizes of bands obtained are given to the right of each panel.

The retina from a number of mammalian species was dissected into neural retina and RPE and used for RNA isolation. As show in Figure 1A, both retinal tissues were found by RT-PCR to contain the α-synuclein mRNA in all the species analyzed. At the protein level, α-synuclein expression was also detected by immunoblotting in rodent (mouse and rat), bovine, and primate neural retinas, where it migrated with an (anomalous) apparent molecular mass of 19 kDa (Figure 1B), consistent with previous reports [1,6]. Expression in the RPE of the α-synuclein protein was also evidenced for the bovine, monkey (Figure 1B) and mouse (data not shown) retinas.

**Figure 2:**
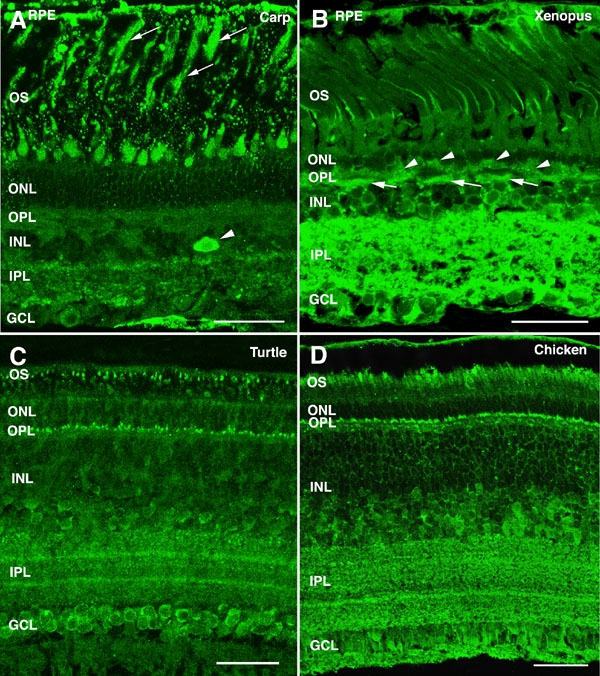
α-Synuclein immunoreactivity pattern in the retina of non-mammalian vertebrates. Shown are retinal sections from (**A**) carp, (**B**) Xenopus, (**C**) turtle, and (**D**) chicken immunolabeled for α-synuclein. Arrows point to rod outer segments in panel **A** and to horizontal cells in panel **B**. Arrowheads point to amacrine cells in panel **A** and to photoreceptor axon terminals in panel **B**. The following abbreviations were used: outer nuclear layer (ONL), ganglion cell layer (GCL), inner nuclear layer (INL), outer plexiform layer (OPL), inner plexiform layer (IPL), retinal pigment epithelium (RPE), and outer segments (OS). Each bars equals 40 μm.

The α-synuclein distribution profile in the retina was examined by means of immunofluorescent confocal microscopy in a wide variety of vertebrate species. A fairly-consistent pattern of α-synuclein retinal distribution was found across non-mammalian vertebrates, including fish, amphibians, reptiles, and birds, as illustrated in the low-magnification views of immunostained retinal sections shown in Figure 2. In the carp, photoreceptor cells were found to contain high levels of α-synuclein in their outer segments (OS; Figure 2A). In addition, a strong immunoreactivity was observed in a certain subtype of amacrine cells (Figure 2A, arrowhead), whose fine dendritic processes ramified in the inner plexiform layer (IPL). Additionally, some ganglion cells showed a weaker α-synuclein immunostaining. In Xenopus the strongest expression of this protein was found in the IPL (Figure 2B), although immunoreactive cell bodies were also visible in the inner nuclear layer (INL). Here horizontal cells (Figure 2B, arrows) were identified by their location at the outermost part of the INL, close to the outer plexiform layer (OPL), and their characteristic thick horizontal processes, whereas α-synuclein-positive rows of cells in the innermost part of the INL should correspond to amacrine cells. Neurons lying in between these two cell types could be ascribed to bipolar cells. Similar to fish, a number of ganglion cells in the toad displayed α-synuclein immunoreactivity as well, although photoreceptors were not labeled with antibodies to α-synuclein except in their axon terminals in the OPL (Figure 2B, arrowheads). In the turtle, two immunoreactive bands were evident in the outer retina, one corresponding to the OS of photoreceptors and the other to their synaptic endings in the OPL (Figure 2C). α-Synuclein was also present in cell bodies residing in the innermost rows of the INL, likely corresponding to bipolar and amacrine cells. The IPL was also labeled for α-synuclein with two bands of intense immunostaining observable in strata S2 and S4. Most cells in the ganglion cell layer also stained positive for this protein (Figure 2C). As with the turtle, two bands of α-synuclein immunoreactivity in the chicken were detected in the outermost neural retina, one corresponding to the photoreceptor OS and the other to the OPL (Figure 2D). Additional α-synuclein expression was evident in the INL within the somata of bipolar and amacrine cells. Two strongly-immunopositive bands were visible in the IPL strata S2 and S4. Finally, α-synuclein was found to be present as well in the cell bodies and axons of some ganglion cells (Figure 2D).

**Figure 3:**
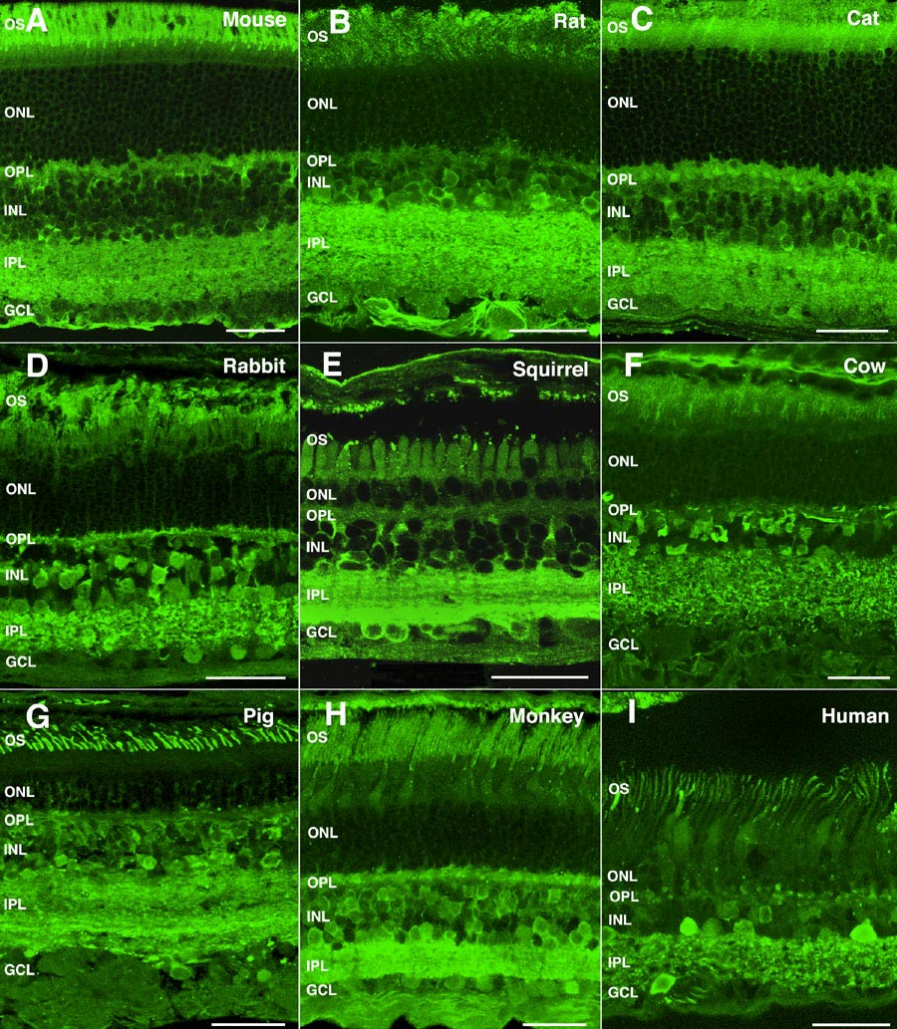
α-Synuclein immunoreactivity pattern in the retina of mammalian vertebrates. Shown are retinal sections immunolabeled for α-synuclein from (**A**) mouse, (**B**) rat, (**C**) cat, (**D**) rabbit, (**E**) squirrel, (**F**) cow, (**G**) pig, (**H**) monkey, and (**I**) human. The following abbreviations were used: outer nuclear layer (ONL), ganglion cell layer (GCL), inner nuclear layer (INL), outer plexiform layer (OPL), inner plexiform layer (IPL), and outer segments (OS). Each bars equals 40 μm.

The α-synuclein expression pattern was also analyzed by immunohistochemistry in the retina of a number of mammalian species ranging from rodents to humans. High levels of α-synuclein were found in the photoreceptor OS layer of mammals of nocturnal vision bearing rod-dominant retinas, such as rodents. A faint immunoreactive band was also observed in the OPL of mouse (Figure 3A) and rat (Figure 3B) retinas. α-Synuclein was present as well in bipolar and amacrine cells, and a homogeneous pattern of intense immunolabeling was evident through the whole thickness of the IPL, without observable stratification. Weak immunoreactivity was additionally seen in mouse and rat ganglion cells. In the cat retina, α-synuclein labeling was observed in the OS of photoreceptors and in their synaptic endings in the OPL (Figure 3C). As observed in the mouse and rat, bipolar and amacrine cell bodies of the cat retina were also immunostained whose dendrites stratified at all sublevels (S1 through S5) of the IPL, and some ganglion cells were also α-synuclein-immunoreactive. A similar α-synuclein distribution pattern was found in the rabbit (Figure 3D), an animal with both crepuscular and nocturnal vision capabilities. In the cone-dominant retina of the squirrel (Figure 3E), a diurnal-vision mammal, a strong α-synuclein labeling was detected in photoreceptor OS, and a cytoplasmic staining was apparent in the somata of several cell types lying in the INL, including bipolar, amacrine and likely also horizontal cells. α-Synuclein expression was prominent in the IPL of this species, with a band of lower immunoreactivity seen in stratum S3, along with some immunolabeled ganglion cells. The cow (Figure 3F) and pig (Figure 3G), exhibited significant α-synuclein levels in photoreceptors only in their OS, together with a weak staining in the OPL. α-Synuclein immunostaining was also found in amacrine and bipolar cell bodies in the INL and in processes spreading throughout the IPL. Similar patterns of distribution of this protein were observed in the retina of primates, such as the macaque (Figure 3H) and the human (Figure 3I).

**Figure 4:**
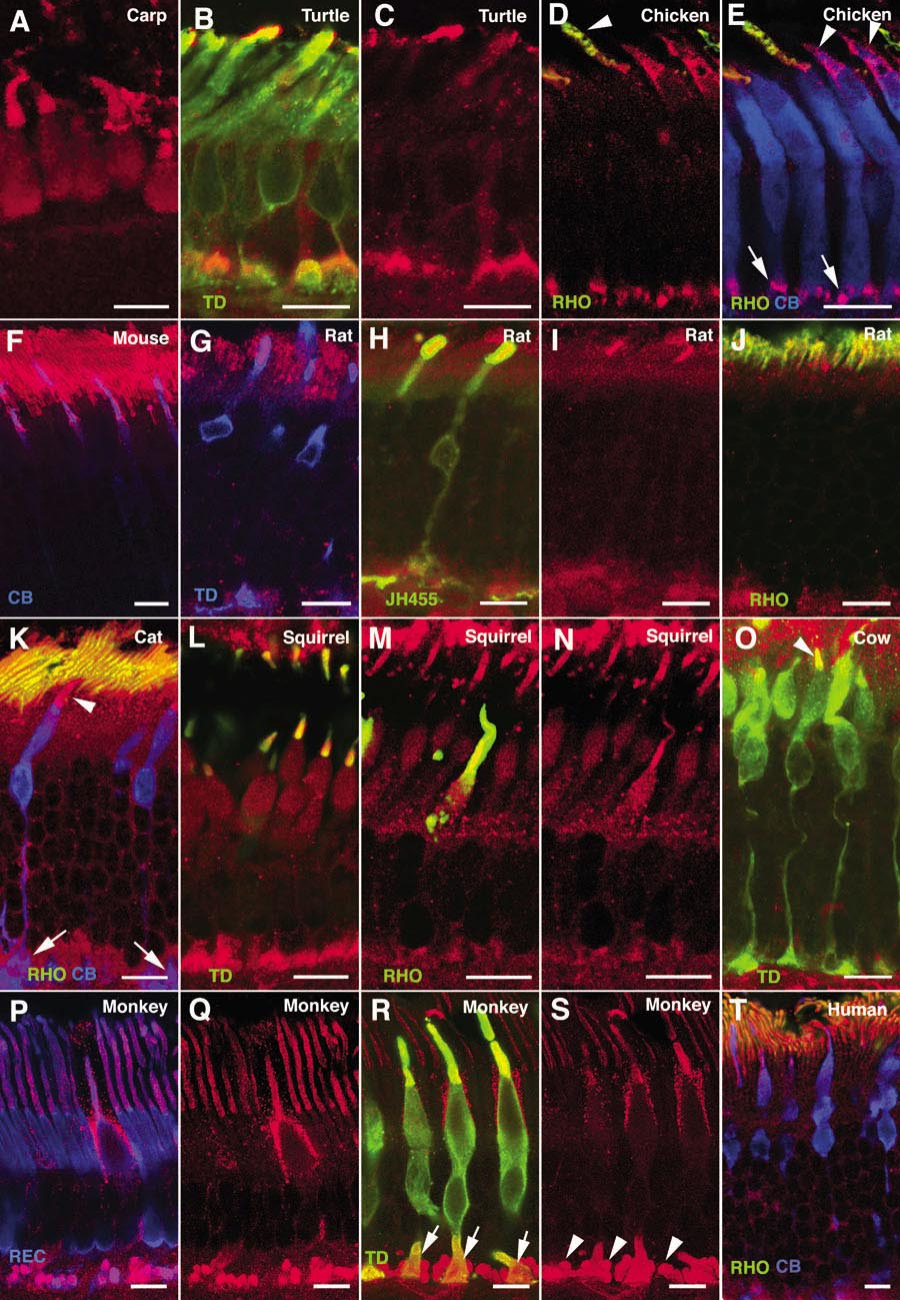
Expression of α-synuclein in photoreceptor cells. Shown are immunolabeled views of (**A**) carp, (**B**, **C**) turtle, (**D**, **E**) chicken, (**F**) mouse, (**G**-**J**) rat, (**K**) cat, (**L**-**N**) squirrel, (**O**) cow, (**P**-**S**) monkey, and (**T**) human photoreceptors. α-Synuclein immunostained red in all panels. Single, double, or triple labelings for different photoreceptor markers are shown. Cone transducin (TD) stained green in panels **B**, **L**, **O**, and **R**, and stained blue in panel **G**. Rhodopsin (RHO) stained green in panels **D**, **E**, **J**, **K**, **M**, and **T**, and S-cone opsin (JH455) stained green in panel **H**. Calbindin (CB) stained blue in panels **E**, **F**, **K**, and **T**, as did recoverin (REC) in **P**. Arrowheads point to outer segments of rods (panel **D**) and cones (panels **E**, **K**, **O**). Arrows mark cone pedicles (panels **E**, **K**, **R**), while arrowheads point to rod spherules (panel **S**). Each bars equals 10 μm.

To further address the intracellular distribution of α-synuclein in vertebrate photoreceptors, we performed double and triple immunostainings with antibodies to α-synuclein and to specific molecular markers for cones and rods. Micrographs of the light-adapted carp retina, where all cones appear shortened in length and rods elongated, are presented in Figure 4A, showing the presence of this protein in the cone OS. Figure 2A, illustrates α-synuclein immunoreactivity in the rod OS of the fish (arrows). A double immunolabeling of the turtle retina for α-synuclein and cone transducin is shown in Figure 4B, together with staining for α-synuclein alone in Figure 4C. As seen, colocalization between these two markers was detected in this reptile in both the OS and pedicles of cones. In the chicken, double staining for α-synuclein and rhodopsin revealed the presence of α-synuclein in the OS of rods (Figure 4D, arrowhead), whereas triple labeling for α-synuclein, rhodopsin, and calbindin, illustrated in Figure 4E, showed colocalization of α-synuclein and calbindin in the cone OS (arrowheads) and axon terminals (arrows). Expression of α-synuclein in the rod OS of the mouse, scotopic retina is shown in Figure 4F (red), and double labeling with calbindin showed colocalization between these two markers in the OS of cones (magenta). Colabeling for α-synuclein and transducin was also found in the cone OS of the rat retina (Figure 4G, magenta). To determine whether blue cones in particular expressed α-synuclein, coimmunolabeling was carried out with antibodies to S-cone opsin (JH455). Colocalization between these two proteins was observed in the OS of blue cones (compare Figure 4H,I). Double staining for α-synuclein and rhodopsin also revealed that rod photoreceptors of the rat exhibited α-synuclein in their OS (Figure 4J). In the cat, triple immunostaining for α-synuclein, calbindin and rhodopsin disclosed an intense colocalization of α-synuclein with rhodopsin in the OS of rods, as shown in Figure 4K (yellow), and with calbindin in the OS (arrowhead) and pedicles (arrows) of cones. As for the cone-dominant retina of the squirrel, α-synuclein colabeling with transducin was found in the OS of cones (Figure 4L, yellow), and with rhodopsin in the OS of rods (Figure 4M, yellow). α-Synuclein was contained as well in the axon terminals of photoreceptors in this species (Figure 4L-N). The presence of this protein in cone OS of the bovine retina is shown by its colabeling at this location with transducin (Figure 4O, arrowhead). For primates, we used recoverin as a simultaneous marker for cones and rods. As illustrated in the macaque retina (compare Figure 4P,Q), α-synuclein located to the OS of both photoreceptor cell types (Figure 4P, magenta). A double labeling with cone transducin revealed its additional presence in cone pedicles (Figure 4R, arrows) and rod spherules (Figure 4S, arrowheads), the latter being transducin-negative. A pattern of α-synuclein distribution similar to that observed in the monkey was found in human photoreceptors upon triple immunostaining for α-synuclein, calbindin and rhodopsin (Figure 4T).

**Figure 5:**
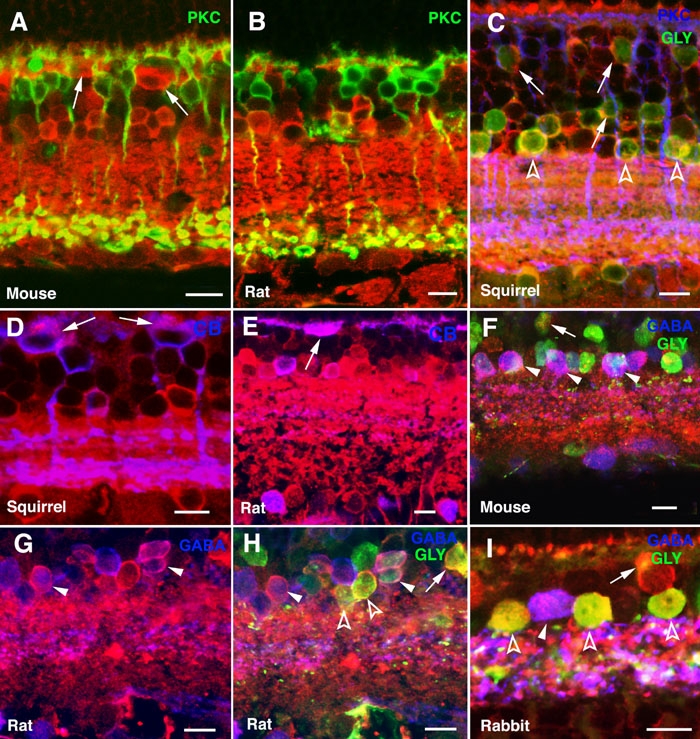
Expression of α-synuclein in horizontal, bipolar, and amacrine cells. Shown are immunolabeled sections of (**A**, **F**) mouse, (**B**, **E**, **G**, **H**) rat, (**C**, **D**) squirrel, and (**I**) rabbit retinas. α-Synuclein immunostained red in all panels. Single, double, or triple labelings were carried out for the indicated retinal neuronal markers. Protein kinase C (PKC) stained green in panels **A** and **B** and blue in **C**. Glycine (GLY) stained green in panels **C**, **F**, **H** and **I**. Calbindin (CB) stained blue in panels **D** and **E**, as did GABA in panels **F**-**I**. Arrows point to horizontal cells in **A**, **D** and **E**, and to glycinergic bipolar cells in panels **C**, **F**, **H**, and **I**. Open arrowheads point to glycinergic amacrines (panels **C**, **H**, **I**) and closed arrowheads to GABAergic amacrines (panels **F**-**I**). Each bars equals 10 μm.

With the aim of assessing the particular cell subtypes expressing α-synuclein among the neurons making synapses in the two retinal plexiform layers, we performed double and triple immunolabeling experiments for α-synuclein and specific markers for bipolar, horizontal, and amacrine cells. As shown for the mouse (Figure 5A) and rat (Figure 5B) retinas, the distribution of the α-synuclein protein in the IPL was homogeneous, i.e. without noticeable stratification along this layer. Double staining with antibodies to α-synuclein and to protein kinase C (PKC) α isoform, a marker for rod bipolar cells, showed that α-synuclein was present in the axon terminals of this cell type in the IPL (Figure 5A,B, yellow), with additional weak immunoreactivity observed in their dendritic terminals in the OPL. Immunolabeling for PKC in the squirrel retina has been reported in rod bipolar cells of the B4 subtype, whose axons ramify in the IPL stratum S5, and in cone bipolars identified as belonging to the B5 and B6 subtypes, whose axon terminals locate to strata S3 and S4 [36]. Figure 5C shows colocalization of α-synuclein and PKC in bipolar cells of the B4, B5, and B6 subtypes in this species. On the other hand, anti-calbindin antibodies, which stain cone bipolar cells in the squirrel retina, allowed the identification of three cone bipolar subtypes according to their branching pattern [36] which exhibited α-synuclein in their axon terminals: the B2 subtype, whose dendrites stratify in stratum S2 of the IPL, the B5 sybtype, branching at the IPL S4-S5 interphase, and the B8 subtype, bistratifying at the S1-S2 and S4-S5 borders (Figure 5D). In some mammalian species horizontal cells were also labeled with antibodies to α-synuclein (Figure 5A, arrows). As shown, colocalization between calbindin and α-synuclein was found in horizontal cells of the squirrel (Figure 5D, arrows) and the rat (Figure 5E, arrow). To determine whether α-synuclein positive cells in the INL used either GABA glycine, or both as neurotransmitters, we did triple immunolabelings for these markers. Some GABAergic amacrine cells were found to exhibit α-synuclein immunoreactivity, as shown for the mouse (Figure 5F), rat (Figure 5G,H), and rabbit (Figure 5I) retinas (closed arrowheads). In addition, colocalization between α-synuclein and glycine was observed in amacrine cells of the squirrel (Figure 5C), rat (Figure 5H), and rabbit (Figure 5I) retinas (open arrowheads). Some α-synuclein-positive amacrines were found to contain both GABA and glycine as neurotransmitters (Figure 5F, white cells). Finally, some glycinergic cone bipolar cells in these species were detected as well to express α-synuclein (Figure 5C,F,H,I, arrows).

**Figure 6:**
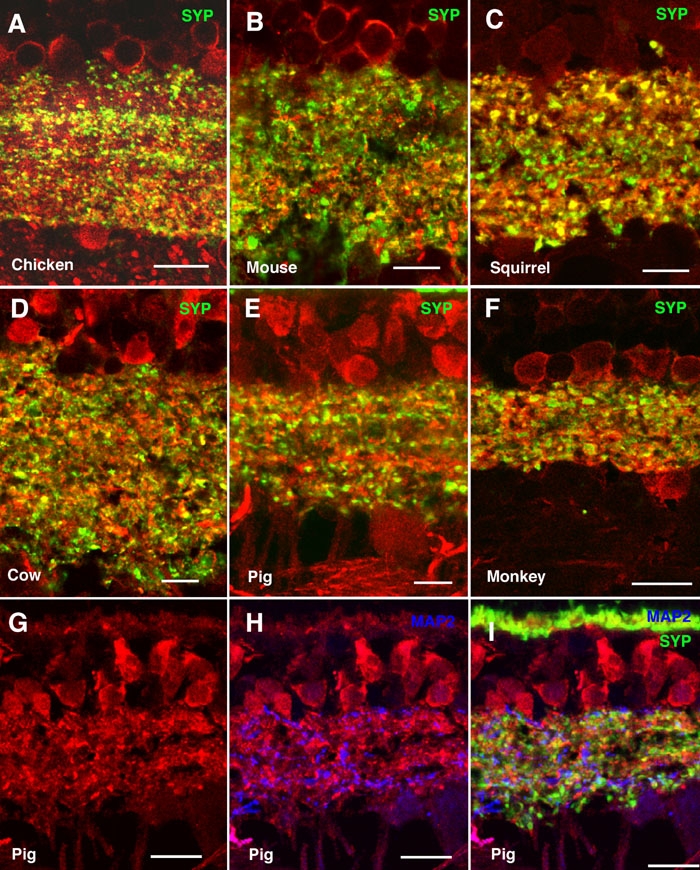
Colocalization between α-synuclein and synaptophysin in the inner plexiform layer. Shown are immunolabeled retina sections of (**A**) chicken, (**B**) mouse, (**C**) squirrel, (**D**) cow, (**E**, **G**-**I**) pig, and (**F**) monkey. α-Synuclein immunostained red in all panels, synaptophysin (SYP) stained green in panels **A**-**F** and **I**, and microtubule-associated protein-2 (MAP2) stained blue in panels **H** and **I**. Lack of colocalization between α-synuclein and synaptophysin is evident in panel **I**. Each bar equals 10 μm.

Synaptophysin is a membrane protein constituent of presynaptic vesicles in the CNS, including the retina [37], which is present in photoreceptor axon endings in the OPL and in bipolar and amacrine cell terminals in the IPL [38,39]. Double labeling for α-synuclein and synaptophysin showed colocalization in many, but not all, immunoreactive puncta in the IPL of all the species studied, from birds to primates (Figure 6A-F, yellow). This pattern was attributed to the existence of different bipolar and amacrine cell subtypes that contained either or both proteins in their synaptic terminals. Figure 6I illustrates the expected colocalization between α-synuclein and synaptophysin in the OPL for the pig retina.

To determine whether α-synuclein was present in the dendrites of ganglion cells, we performed double immunostaining experiments for α-synuclein and MAP2, a specific marker for this retinal neuronal type. Figure 6G shows the distribution of α-synuclein in the IPL of the pig retina. Double labeling with antibodies to α-synuclein showed the lack of a clear colocalization of this protein with MAP2 (Figure 6H), indicating that ganglion cells are not likely to express α-synuclein in their dendrites in the pig retina. Further, no colocalization was found between MAP2 and synaptophysin in the dendrites of ganglion cells in the IPL (Figure 6I).

**Figure 7:**
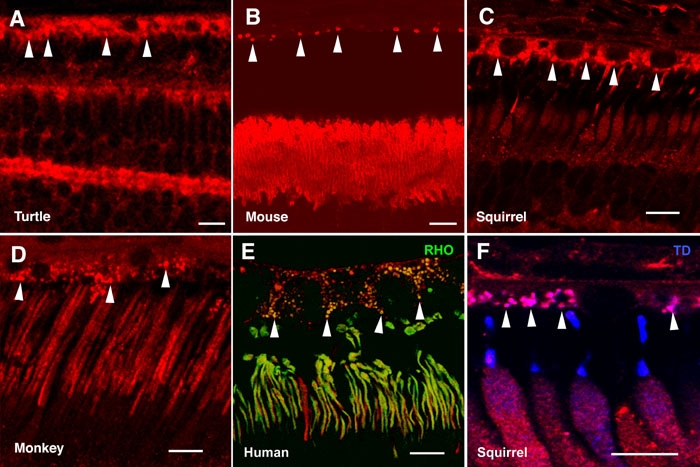
Expression of α-synuclein in retinal epithelial cells. Shown are immunolabeled retina sections of retinal pigment epithelium and photoreceptor outer segment layers for (**A**) turtle, (**B**) Swiss mouse, (**C**, **F**) squirrel, (**D**) monkey, and (**E**) human retinas. α-Synuclein was immunostained red in all panels, rhodopsin (RHO) stained green in panel **E**; and cone transducin (TD) stained blue in panel **F**. Arrowheads point to phagosome-like structures in the apical region of retinal epithelial cells. Each bars equals 10 μm.

Given our finding of α-synuclein mRNA and protein in the mammalian RPE (Figure 1), we analyzed α-synuclein subcellular distribution in this retinal layer in vertebrates by immunohistochemical methods. A cytoplasmic labeling pattern was found in RPE cells of vertebrates, especially in carp (Figure 2A), toad (Figure 2B), turtle (Figure 7A), squirrel (Figure 7C), and monkey (Figure 7D). α-Synuclein appeared excluded from the nuclei of these cells, and a strong punctate immunoreactivity pattern was observed in the region of epithelial cells close to the OS of cones and rods (Figure 7, arrowheads). A double immunostaining for α-synuclein and rhodopsin revealed a number of such structures colocalizing both proteins in the human retina (Figure 7E, yellow). This was also the case for α-synuclein and cone transducin in the squirrel retina (Figure 7F, magenta), which, based on their size (0.7-1.4 μm), could well constitute phagosomes containing OS disc membranes being phagocytized by RPE cells.

## Discussion

The synucleins constitute a family of structurally-related proteins of widespread expression in the vertebrate neurons. Dysfunction of these proteins has been linked to the onset and progression of neurodegenerative diseases [7,8,10,21,22,40]. Members of this family include α- and β-synucleins, which locate at axon presynaptic terminals of the CNS, and γ-synuclein, which is most abundant in neurons of the peripheral nervous system but is also present in the retina and optic nerve. Although their precise functions remain not completely understood, synucleins are throught to integrate presynaptic signaling and membrane trafficking [7,9,15]. The pattern of expression and distribution of α-synuclein in the retina has not been previously characterized, and only its predominant presence in the IPL of the mouse and human retinas has been shown [41,42]. However, the particular retinal cell types exhibiting α-synuclein in the mammalian retina are not known, and its distribution pattern in the retina of other vertebrates has not been previously addressed.

In this work we showed expression of α-synuclein at the mRNA and protein levels in the neural retina of mammals, including rodent (rat and mouse), bovine, and primate (human and monkey) retinas. This protein exhibited an apparent size of 19 kDa on immunoblots, despite its real 14.5 kDa molecular mass, as previously reported [1,2,4,6,16,23]. This variation, leaving aside possible postranslational modifications, has been mainly attributed to low binding of SDS by its highly-acidic C-terminus [6,43].

The retinal distribution profile of α-synuclein, as assessed by immunohistochemistry, was consistent in all the species studied, which was indicative of a similar role for this protein across vertebrates. In this pattern, we have found a strong α-synuclein immunolabeling in the OS of both cone and rod photoreceptors, a location where α-synuclein has been previously not noticed [41,42]. Since roles have been proposed for α-synuclein in the formation and recycling of synaptic vesicles, as well as in the maintenance of synapse structure in the CNS [7-10,15], and given that OS discs are formed within photoreceptors through the apposition and fusion of membrane vesicles, an involvement of α-synuclein in the turnover of OS discs and/or in maintenance of their structural integrity can be postulated.

To date, the presence of α-synuclein in the OPL of the mammalian retina has not been reported. However, we observed a concentration of α-synuclein at photoreceptor axon terminals of vertebrates, including both cone pedicles and rod spherules. Additionally, we noted several subtypes of bipolar and amacrine cells of retinas from both nocturnal and diurnal animals that were found to express α-synuclein. This protein was immunolocalized to the cell bodies and (predominantly) to the axon terminals of both rod and cone bipolar cells, as well as to the somata and dendrites of both glycinergic and GABAergic amacrine cells, yielding an intense immunoreactivity pattern along the IPL. Given that α-synuclein accumulates at presynaptic nerve endings in the brain, where it likely participates in the synaptic-vesicle cycle, including neurotransmitter storage, release and reuptake [13,14,44-46], an equivalent role of α-synuclein in synaptic function at both plexiform layers of the vertebrate retina can thus be inferred.

Synaptophysin is a synaptic-vesicle transmembrane protein with an active involvement in the regulation of vesicular exocytosis in the CNS. This protein has been immunolocalized to presynaptic terminals at both the OPL and IPL in the retinas of a wide range of vertebrate species [38,39,47-50], where it is a constituent protein of synaptic vesicles [37,48,51]. We found colocalization of α-synuclein with synaptophysin in photoreceptor terminals in the OPL as well as in bipolar and amacrine cell endings in the IPL, although no such colocalization was observed in the dendrites of ganglion cells. These results indicate that α-synuclein is present in presynaptic, but not postsynaptic, terminals of retinal neurons in both plexiform layers, where it could be associated with synaptic vesicles to modulate neurotransmission.

Expression of α-synuclein was not restricted to the neural retina, but this protein was also present at significant levels in the RPE. Although γ-synuclein has been found in cultured bovine RPE cells [52], α-synuclein has been reported to be absent from the human RPE, iris, cornea and lens [41]. However, we have determined by immunohistochemistry that α-synuclein expression in the RPE is not just a feature of mammals, it extends to vertebrates in general. In addition to its general cytoplasmic distribution, an accumulation of α-synuclein was apparent in the form of a punctate pattern in the apical region of RPE cells. The size of these aggregates and the colocalization of α-synuclein in these particles with rhodopsin or transducin, which are proteins contained in rod and cone OS, respectively, strongly suggest that they constitute phagosomes [53], i.e. vesicular structures arising from the phagocytosis by retinal epithelial cells of discs shed from the OS of photoreceptors. Therefore, α-synuclein detected in these structures within RPE cells would derive from its presence in photoreceptor disc membranes, a possibility that should be ascertained by electron microscopy analysis. However, our findings of abundant α-synuclein mRNA levels in RPE tissue along with α-synuclein protein distribution throughout the cytoplasm indicate that retinal epithelial cells exhibit as well the property of α-synuclein mRNA and protein synthesis. In this context, it must be mentioned that α-synuclein expression has also been reported at low levels in organs other than the brain [1,54], as well as in epithelial cells of the human olfactory mucosa [55].

Taken together, our results allow us to postulate a function of α-synuclein in synaptic transmission through both ribbon-type and conventional synapses of the retina. Such an involvement of this protein has been previously suggested at the IPL level [41]. Given its wide retinal distribution and potentially relevant role in retinal function, it remains a formal possibility that, inasmuch as certain mutations in the *SNCA* gene are known to be associated with neurological disorders in the brain, unsought α-synuclein genetic alterations could be causative of retinitis pigmentosa and other retinal neurodegenerative diseases. In this context, retinal degeneration has been found in parkinsonian *Drosophila* transgenic lines expressing wild-type or mutant variants of human α-synuclein in the eye [56]. This issue deserves further study.
